# Poor Sleep during the First Peak of the SARS-CoV-2 Pandemic: A Cross-Sectional Study

**DOI:** 10.3390/ijerph18010306

**Published:** 2021-01-04

**Authors:** Stefania Costi, Sara Paltrinieri, Barbara Bressi, Stefania Fugazzaro, Paolo Giorgi Rossi, Elisa Mazzini

**Affiliations:** 1 Scientific Directorate, Azienda Unità Sanitaria Locale-IRCCS di Reggio Emilia, 42123 Reggio Emilia, Italy; stefania.costi@unimore.it; 2 Department of Medicine, Surgery, Dentistry and Morphological Sciences, Università di Modena e Reggio Emilia, 41124 Modena, Italy; 3 Physical Medicine and Rehabilitation Unit, Azienda Unità Sanitaria Locale-IRCCS di Reggio Emilia, 42123 Reggio Emilia, Italy; barbara.bressi@ausl.re.it (B.B.); stefania.fugazzaro@ausl.re.it (S.F.); 4 Department of Biomedical, Metabolic and Neural Sciences, Università di Modena e Reggio Emilia, 41124 Modena, Italy; 5 Epidemiology Unit, Azienda Unità Sanitaria Locale-IRCCS di Reggio Emilia, 42123 Reggio Emilia, Italy; paolo.giorgirossi@ausl.re.it; 6 Medical Directorate, Azienda Unità Sanitaria Locale-IRCCS di Reggio Emilia, 42123 Reggio Emilia, Italy; elisa.mazzini@ausl.re.it

**Keywords:** quarantine, sleep, pandemics, lifestyle, SARS-CoV-2, anxiety

## Abstract

The restrictions enacted during lockdown to limit the spread of the novel coronavirus (SARS-CoV-2) have led to changes in people’s lifestyle habits. In Italy, these restrictions have dramatically changed the way people work and spend their leisure time, also with repercussions on diet and physical activity. An anonymous survey was disseminated via websites and social media to a convenience sample of the Italian population during and immediately after the first lockdown (10 March–18 May 2020). Data collected on 1826 individuals show that lockdown might have worsened the quality of sleep of almost half of the participants in this cross-sectional study. This worsening was associated with a deterioration in crucial determinants of health, such as physical activity and diet (OR 1.68; 95% CI 1.18–2.40 and OR 4.19; 95% CI 2.51–6.96, respectively), with symptoms of psychological distress, such as tension (OR 3.88; 95% CI 2.74–5.52) and loneliness (OR 3.27; 95% CI 2.23–4.79), and with the presence of financial problems (some OR 1.86; 95% CI 1.27–2.72; many OR 7.27; 95% CI 3.59–14.73). The multivariate regression analysis models confirmed these associations. This impact on sleep quality was seen especially among females, those with low education level, and those who experienced financial problems.

## 1. Introduction

The current pandemic of severe acute respiratory syndrome coronavirus 2 (SARS-CoV-2) has placed the world population under enormous stress. Large geographical areas and the resident populations have been forced to undergo periods of lockdown to halt the spread of the virus and to limit the impact on healthcare systems. In March 2020, the Italian Government imposed two months of emergency lockdown, with containment measures unheard of in the national territory, sometimes characterized by mandatory quarantine [1]. These measures included the closing of businesses, schools and universities, and the banning of all recreational outdoor activities; the only exceptions were the provision of essential services for health and basic needs (i.e., food, municipal services, etc.). The lockdown radically altered the population’s lifestyle, and several containment measures lasted for weeks beyond the end of the full lockdown period: many people saw their work temporarily suspended, others were forced to work or study from home, and everybody was subjected to mobility restrictions and were obliged to cease any non-essential activity. While these measures were essential to reducing outbreaks, they may have led to the adoption of unhealthy behaviours (e.g., more sedentary lifestyle, changes in diet) [2]. Further, these strong limitations on personal freedom could have led to increased levels of psychological distress in the population [3] caused by the fear of getting sick, the fear of experiencing financial problems, the increased tension in households, as well as the isolation from elderly family members, who are at greater risk of death.

All these factors could have worsened the quality of sleep, defined as one’s satisfaction with the sleep experience, which integrates aspects of sleep initiation, sleep maintenance, sleep quantity, and refreshment upon awakening [4]. Unsatisfactory sleep can lead to short and long-term harmful psychological and psychosocial effects on health [5,6]. Thus, this observational study aimed to investigate the quality of sleep in a convenience sample of Italians under lockdown during the first epidemic peak of the COVID-19 pandemic. We also investigated the associations between the quality of sleep and sociodemographic characteristics, changes in lifestyle, and the presence of psychological distress. 

## 2. Materials and Methods

This cross-sectional study was endorsed by the Local Health Authority (LHA) of the Province of Reggio Emilia (Italy), with a population of 533,158; 66% of the population is between the ages of 18 and 70. This province was one of the most affected by the first epidemic peak of COVID-19 in Italy, with a daily incidence rate of about 8/1000 inhabitants during lockdown (10 March–18 May 2020) [7].

An online survey investigating lifestyle habits and any changes that occurred during lockdown was developed by two epidemiologists, two physiotherapists, and one occupational therapist. The survey did not collect personal data and was completely anonymous as to the source, which is why the consulted Local Ethics Committee deemed approval unnecessary. However, the survey underwent an ethics review conducted by an expert in the field; after approval, it was disseminated online to individuals living in the Province of Reggio Emilia. The survey was advertised through the websites and social media of the LHA, the major municipalities of the province, the network of the municipal pharmacies, and the local patient associations that joined the initiative. Citizens could access the survey without restrictions and in complete anonymity from 4 May 2020 until 15 June 2020. 

The survey (Supplementary Material File S1) explored the following areas: (a) sociodemographic data (14 questions); (b) work-related data (5 questions); (c) use of digital devices (3 questions); (d) lifestyle and general health (23 questions); (e) use of local social support services (3 questions); and (f) symptoms of psychological distress, i.e., the sense of tension, upset, worry, fear, loneliness, and/ or uncertainty (one question).

Apart from two open-ended questions, the answers were multiple-choice. In some cases, more than one choice was possible. The survey took an average of 15 min to complete. 

The main outcome of this cross-sectional study was to describe the quality of sleep during and immediately after lockdown, investigated through the following two questions: “Have your sleep habits changed since the beginning of lockdown (amount and/or regularity of sleep)?” and “How do you rate your sleep quality now”?

We also searched for associations between sleep quality categories and sociodemographic and occupational characteristics, changes in lifestyle (physical activity, eating habits, smoking, and alcohol consumption), and psychological distress.

### 2.1. Data Privacy and Consent for Participation

During the informed consent process, participants were informed that all data would be anonymized at the source and would be used exclusively for research purposes. By completing the survey, participants voluntarily consented to participate in this study. As response would be saved only by clicking the “submit” button, that participant could stop participating in the study at any stage before submission, and their responses would not be saved. As the survey was disseminated to the local community, participants were not asked to provide name, date of birth, residency, and/or job to ensure complete anonymity. 

### 2.2. Statistical Analysis

Participants who completed the survey were categorized based on the dependent variable, i.e., their self-reported quality of sleep. They were grouped by sex, age class, education level, household composition, any financial problems, occupational status, lifestyle habits, and psychological distress. We report both the number and proportion of missing values. We looked for associations (crude odds ratio—OR—and its 95% confidence interval—95% CI) between the sociodemographic and occupational characteristics, changes in lifestyle, and the presence of psychological distress as independent variables and the quality of sleep categories. Separate models of multivariate regression analysis, all adjusted for age, sex, and education level, were built for any of the independent variables which, based on the crude OR and its 95% CI, proved to be strongly associated with quality of sleep. Missing values were not included in the analyses. Statistical analyses were performed using jamovi 1.2.27 (Jamovi Project, Sydney, Australia) [8]. 

## 3. Results

Overall, 1826 citizens in the Province of Reggio Emilia participated in the study. Most were female (76.5%), aged 18 to 64 years (88.7%), with medium-high education level (92.8%), and with no financial problems (72.3%). Most lived with one or more individuals (88.6%) and continued to work (in person or remote) during lockdown (69.8%) (Table 1). 

At the time of the survey, most participants reported good sleep quality (*n* = 1026; 56.2%), with 39.5% (*n* = 721) reporting poor sleep quality. However, 53.7% of participants experienced a change in their sleep habits since the beginning of lockdown, more frequently represented by a decrease in the amount and/or regularity of sleep (OR 3.68; 95% CI 2.32–5.82; *p* < 0.001).

About one third of participants reported that their physical activity habits worsened (35.1%), while far fewer reported an improvement (5.3%). Most participants reported that their eating habits had changed: about one third reported an improvement (33.5%), i.e., all the self-reported changes went in the direction of a healthier diet (e.g., eating more fruit and vegetables, regular meals, etc.); others reported a worsening (17.6%), i.e., all changes went in the direction of a less healthy diet (e.g., more prepackaged foods, carbonated drinks), and 18.5% of participants had both changes in the direction of healthier and less healthy diet. Alcohol consumption changed for an overlapping number of participants (increased or decreased, 12.5% and 12.7%, respectively), and lower proportions of participants reported that their smoking habits had increased or decreased (7.7% and 4.1%, respectively).

More than half of participants experienced feelings of uncertainty (54.5%). Moreover, a considerable proportion of individuals experienced emotions of worry (44.4%), upset (20.4), tension (19.7%), fear (16.6%) and loneliness (13.6%). 

Male sex seemed to be associated with better sleep quality (OR 0.47; 95% CI 0.29–0.76), as was a high education level compared to low (OR 0.44; 95% CI 0.23–0.81). Factors more strongly associated with poor sleep quality were the presence of some or many financial problems (OR 1.86; 95% CI 1.27–2.72 and OR 7.27; 95% CI 3.59–14.73, respectively), a change toward less physical activity or worse eating habits (OR 1.68; 95% CI 1.18–2.40 and OR 4.19; 95% CI 2.51–6.96, respectively), and perceiving feelings of psychological distress, such as tension (OR 3.88; 95% CI 2.74–5.52), loneliness (OR 3.27; 95% CI 2.23–4.79), upset (OR 3.26; 95% CI 2.29–4.64) uncertainty (OR 2.89; 95% CI 1.93–4.31), fear (OR 2.74; 95% CI 1.89–3.97), and/or worry (OR 2.50; 95% CI 1.75–3.58). A change toward mixed behaviour in eating habits was also associated with poor sleep quality.

The multivariate regression analysis models confirmed the association between poor sleep quality and the presence of some or many financial problems (OR 1.74; 95% CI 1.17–2.57 and OR 6.96; 95% CI 3.32–14.55, respectively) and less physical activity or worse eating habits (OR 1.62; 95% CI 1.13–2.32 and OR 3.7; 95% CI 2.2–6.24, respectively). Again, the association between a change toward mixed behaviour in eating habits and poor sleep quality was confirmed in the adjusted model of multivariate regression analyses. The regression models also confirmed the association between poor sleep quality and the presence of a sense of tension (OR 3.51; 95% CI 2.44–5.03), loneliness (OR 3.07; 95% CI 2.07–4.53) upset (OR 3.01; 95% CI 2.10–4.32), uncertainty (OR 2.71; 95% CI 1.81–4.06) fear (OR 2.42; 95% CI 1.65–3.54) and/ or worry (OR 2.30; 95% CI 1.60–3.32) (Appendix A).

## 4. Discussion

The results of this study highlight the association between lockdown and poor sleep quality in the Italian population. These results suggest that lockdown might impact sleep quality and that this association could be mediated by crucial determinants of health, such as physical activity and diet, and to a lesser extent, smoking and alcohol consumption, the presence of financial problems, and symptoms of psychological distress. Indeed, anxiety was widespread among the Italian population during lockdown, as were feelings of uncertainty, fear, and loneliness [9,10]. This impact on sleep quality was seen especially among females, those with low education level, and those who experienced financial problems. 

Since the beginning of the pandemic, observational, mostly cross-sectional, studies have been conducted around the world to study lifestyle changes in populations subjected to restrictions. While sleep quality has been frequently investigated, results have not always been consistent. In China, Wang and colleagues found that 75% of 2289 individuals isolated at home rated their sleep as very good [11], but a smaller prospective study found that 37% of a sample of young Chinese adults reported a worsening of their sleep quality during the pandemic [12]. Studies conducted in other countries have demonstrated that lockdown could either lead to an increase in the amount of time spent in bed [13,14], with good sleep efficiency [13] or associated with sleep disorders [15,16] and disruption of one’s habitual circadian rhythm [14]. This topic has also been investigated in Europe, where 55% of Spanish adults changed their sleep pattern after the beginning of the lockdown [17], and individuals who were physically active prior to the SARS-CoV-2 spread reported major sleep problems [18]. In Italy, cross-sectional investigations have suggested that while the number of hours spent in bed might have increased during lockdown [2], many individuals reported insomnia (43%) [19] and poor sleep quality (57%) [9]. Similar percentages of sleep deterioration (48%) were also shown in the UK [20], and a longitudinal study conducted in Italy confirmed the detrimental effects of the 40-day lockdown on all the parameters of the Sleep Quality Index [21], except for sleep duration [22]. Our cohort seems to confirm that sleep quality deteriorated during lockdown, as among those individuals who experienced a change in their sleep quality, 85.8% defined their sleep as poor. This proportion of individuals accounted for 46% of the entire cohort investigated, which is strikingly similar to the proportion of those who reported a worsening of sleep quality in the above-mentioned Italian studies. 

Our results corroborate the findings of previous research that the worsening of sleep quality during lockdown was more pronounced among females [9,23,24]. Poor sleep quality was strongly associated with the presence of many financial problems, although this group in our cohort accounted for a very small proportion of participants (2.1%). However, poor sleep quality was not associated with the interruption of work. One possible explanation for these apparently inconsistent data could be because of the economic measures promptly introduced by the Italian Government to counter the economic effects of the forced closure of most businesses. Similarly, a large survey conducted in the UK found that poor sleep quality during lockdown was associated with perceived financial problems, but not with unemployment [23]. 

In this study, 35.1% of individuals reported a worsening of their level of physical activity, which proved to be a determinant of poor sleep quality in the multivariate analysis. This result was not surprising, as a moderate effect of regular exercise on overall sleep quality is well documented [25,26,27], and the association between less exercise and sleep disorders was confirmed during lockdown in a sample of German individuals [16]. 

In addition, 17.6% of individuals declared a change for the worse in their eating habits, which was associated with poor sleep quality. This proportion of individuals who adopted a less healthy diet is lower than the 35.8% estimated by Di Renzo and colleagues in a cross-sectional survey, which involved 3533 Italians throughout the country [2]. In this regard, it should be noted that 18.5% of our sample introduced changes in their diet that we categorized as mixed, because they were neither completely positive nor completely negative. It is plausible that some of these mixed changes, upon further investigation, could be reclassified as worse. In fact, the association between sleep quality and this type of mixed change in eating habits went in the same direction as that shown by the worsening of one’s diet. Still, regardless of the accuracy of the categorization for this predictive variable, it seems certain that several individuals’ diets worsened and that this was associated with poorer sleep. This also seems to be true outside of Italy, as data collected from more than 1000 individuals in three different continents, including Europe, which found that unhealthy diet increased by 10% during the period of social isolation, and that worse eating habits were significantly associated with a worsening in sleep quality [15].

Finally, our results confirm the association between poor sleep quality and symptoms of psychological distress. The positive relationship between anxiety and poor sleep quality has been extensively investigated and was confirmed during lockdown [9,20,26]. While the cross-sectional design of our study does not allow us to verify whether the symptoms of psychological distress that we documented were related to the restrictions imposed to curtail the spread of the SARS-CoV-2 virus, a causal relationship is plausible, as also suggested by the literature [9,10,15,16,20,28]. The convenience sample investigated was recruited in the Province of Reggio Emilia, one of the most affected Italian provinces during the early phase of the pandemic. Considering that, at the time of the survey, little or nothing was known about the disease and its prognosis, it is plausible that feelings of fear and uncertainty were more widespread in the populations living in areas with a high prevalence of infection [9,29]. Added to this were the concerns arising from the forced temporary closure of many business and industries in one of the most productive areas of Italy and of Europe. The COVID-19 pandemic has called into question the stability of the Italian National Health Service, also triggering uncertainties regarding the continuity of care for thousands of individuals, often elderly and with comorbidities, in one of the longest-lived countries in the world. Therefore, the association between poor sleep quality and feelings of fear and uncertainty was expected.

Less obvious was the relationship between poor quality sleep and feelings of loneliness as, based on the univariate analysis conducted, living alone was not a predictor of poor sleep quality. Thus, loneliness was perceived regardless of the presence of other household members; the reasons underlying this feeling must be sought in other causalities. We have no explanation for this, but it is possible that forced living with others in close quarters for an extended period may have worn down the quality of relationships between individuals, or loneliness may have been due to the forced separation from important, vulnerable family members. Moreover, the sense of loneliness could also have derived from the need to limit interpersonal contact to a minimum, a measure that, while necessary to reduce the contagion, is in stark contrast with Italian culture.

### Limitations and Strengths

The results of this study should be interpreted with caution as they derive from a cross-sectional design, which makes causal inferencing challenging. Moreover, the survey sampling was based on an online invitation, which does not allow for generalization because the population that does not use the Internet was not explored. 

Indeed, a recruitment bias emerged in our sample, as females and working-age individuals with a high education level are overrepresented compared to the general Italian population. While the overrepresentation of females is a feature common to other similar studies [2,9,16,20,28,30], it is likely that the biases observed in age and education reflect a high level of adhesion of participants who are also healthcare professionals or administrative staff of the LHA or of the major municipalities of the Province of Reggio Emilia, which disseminated the survey through their social media and websites. 

Moreover, the outcome of interest and data related to predictive variables were collected through self-reporting rather than through a valid survey or clinical assessment. This was because, as we wanted to capture the changes that occurred during lockdown through a cross-sectional design, we chose specific questions to comply with this purpose. 

Finally, we decided to observe the association between each change occurring during the lockdown, adjusting only for those variables that certainly could not change during the study period and cannot be intermediate effectors of the other putative exposure. However, this approach does not allow us to rule out that some of the observed associations were not independent and could have been due to confounding or could have been mediated by other putative exposures.

Despite these limitations, this study had a relatively high acceptance in our community. The sampling strategy allowed us to collect data from quite a large sample, which in fact numerically mirrors that investigated by other similar study designs conducted to explore the same theme [9,15,28,30]. Further, this was the only sampling method feasible during lockdown. 

## 5. Conclusions

The quality of sleep, one of the determinants of public health, was adversely affected by the lockdown. Our study points out that nearly all individuals who experienced a change in their sleep reported poor quality of the same. This result is plausible, considering the fast disruption of routine caused by the pandemic. In fact, individuals’ well-being may have been seriously affected when both personal and public safety are no longer predictable, regardless of their resilience and their capacity for adaptation in daily life.

We highlighted the effect that sociodemographic and occupational characteristics, changes in lifestyle, and psychological distress have on sleep quality. While this study design does not allow us to determine a causal relationship among potential determinants and poor sleep quality, it nevertheless highlights the presence of categories of people who suffered the most: socially fragile individuals, those reporting negative changes in physical activity and diet, and individuals with psychological distress.

These categories require the attention of policy makers, who should plan strategies to promote healthy lifestyles and strategies to maintain serenity during this difficult period.

Considering that the SARS-CoV-2 infections rate has been increasing since October 2020 and that several restrictions have been reintroduced to contain the spread of the virus, further investigations on this topic should be implemented. For example, studies exploring whether sleep has been affected during this second epidemic peak as well or whether individuals have adapted to the situation and have found strategies to deal with the limitations and uncertainties related to the SARS-CoV-2 spread would be of interested. 

## Figures and Tables

**Table 1:** Odds ratio for quality of sleep during lockdown by sociodemographic characteristics, work-related factors, changes in lifestyle and psychological distress.

			Total		Poor Sleep		Good Sleep		Missing					
			*n*	%	*n*	%	*n*	%	*n*	%				
			1826	100	721	39.5	1026	56.2	79	4.3				
											OR	95% CI		*p*-Value
												Lower	Upper	
Sociodemographic characteristics	Sex	Female	1397	76.5	601	43.0	732	52.4	64	4.6	1.00			
		Male	423	23.2	119	28.1	293	69.3	11	2.6	0.47	0.29	0.76	0.002
		Missing	6	0.3	1	16.7	1	16.7	4	66.7				
	Age	18-44	818	44.8	327	40.0	467	57.1	24	2.9	1.00			
		45-64	802	43.9	336	41.9	438	54.6	28	3.5	0.95	0.67	1.34	0.771
		>65	194	10.6	55	28.4	118	60.8	21	10.8	0.54	0.26	1.11	0.092
		Missing	12	0.7	3	25.0	3	25.0	6	50.0				
	Education level	Low	94	5.1	38	40.4	50	53.2	6	6.4	1.00			
		Medium	805	44.1	329	40.9	432	53.7	44	5.5	0.55	0.30	1.03	0.066
		High	889	48.7	338	38.0	527	59.3	24	2.7	0.44	0.23	0.81	0.009
		Missing	38	2.1	16	42.1	17	44.7	5	13.2				
	Household	Alone	208	11.4	77	37.0	129	62.0	2	1.0	1.00			
		≥1 person	1618	88.6	644	39.8	897	55.4	77	4.8	1.06	0.62	1.79	0.834
	Financial problems	No problems	1320	72.3	464	35.2	794	60.2	62	4.7	1.00			
		Some problems	399	21.9	204	51.1	187	46.9	8	2.0	1.86	1.27	2.72	0.001
		Many problems	38	2.1	23	60.5	15	39.5	0	0.0	7.27	3.59	14.73	<0.001
		Missing	69	3.8	30	43.5	30	43.5	9	13.0				
	Occupational status during lockdown	Continue working	1275	69.8	495	38.8	726	56.9	54	4.2	1.00			
		Activity stopped	181	9.9	82	45.3	96	53.0	3	1.7	1.33	0.80	2.21	0.266
		Retir. Stud. Housew.	240	13.1	86	35.8	143	59.6	11	4.6	0.73	0.42	1.29	0.288
		Unemployed	55	3.0	28	50.9	25	45.5	2	3.6	1.09	0.42	2.81	0.848
		Missing	75	4.1	30	40.0	36	48.0	9	12.0				
Changes in lifestyle	Change in sleep habits	Unchanged	701	38.4	645	92.0	24	3.4	34	4.9	1.00			
		Changed	981	53.7	842	85.8	121	12.3	17	1.7	3.86	2.46	6.05	<0.001
		Uncertain	80	4.4	67	83.8	3	3.8	10	12.5	1.20	0.35	4.10	0.760
		Missing	64	3.5	21	32.8	25	39.1	18	28.1				
	Change in physical activity habits	Unchanged	972	53.2	349	35.9	596	61.3	27	3	1.00			
		Improved	97	5.3	37	38.1	56	57.7	4	4.1	0.59	0.21	1.68	0.330
		Worsen	641	35.1	288	44.9	334	52.1	19	3.0	1.68	1.18	2.40	0.004
		Missing	116	6.4	47	40.5	40	34.5	29	25.0				
	Change in eating habits	Unchanged	530	29.0	152	28.7	361	68.1	17	3.2	1.00			
		Improved	612	33.5	237	38.7	361	59.0	14	2.3	1.49	0.89	2.51	0.125
		Worsen	321	17.6	171	53.3	128	39.9	22	6.9	4.19	2.51	6.96	<0.001
		Mixed behav.	337	18.5	156	46.3	164	48.7	17	5.0	2.41	1.40	4.15	0.001
		Missing	26	1.4	5	19.2	12	46.2	9	34.6				
	Change in alcohol consumption	Unchanged	1275	69.8	496	38.9	726	56.9	53	4.2	1.00			
		Decreased	231	12.7	90	39.0	138	59.7	3	1.3	0.73	0.41	1.28	0.280
		Increased	229	12.5	107	46.7	112	48.9	10	4.4	1.34	0.84	2.13	0.209
		Missing	91	5.0	28	30.8	50	54.9	13	14.3				
	Change in smoking habits	Unchanged	1327	72.7	519	39.1	780	58.8	28	2.1	1.00			
		Decreased	75	4.1	33	44.0	40	53.3	2	2.7	0.79	0.31	2.01	0.628
		Increased	140	7.7	64	45.7	67	47.9	9	6.4	1.39	0.78	2.47	0.251
		Missing	284	15.6	105	37.0	139	48.9	40	14.1				
Psychological distress	Tension	No	1337	73.2	452	33.8	838	62.7	47	3.5	1.00			
		Yes	359	19.7	232	64.6	107	29.8	20	5.6	3.88	2.74	5.52	<0.001
		Missing	130	7.1	37	28.5	81	62.3	12	9.2				
	Upset	No	1284	70.3	444	34.6	804	62.6	36	2.8	1.00			
		Yes	372	20.4	230	61.8	135	36.3	7	1.9	3.26	2.29	4.64	<0.001
		Missing	170	9.3	47	27.6	87	51.2	36	21.2				
	Worry	No	914	50.1	271	29.6	614	67.2	29	3.2	1.00			
		Yes	810	44.4	421	52.0	366	45.2	23	2.8	2.50	1.75	3.58	<0.001
		Missing	102	5.6	29	28.4	46	45.1	27	26.5				
	Fear	No	1404	76.9	501	35.7	847	60.3	56	4.0	1.00			
		Yes	303	16.6	180	59.4	116	38.3	7	2.3	2.74	1.89	3.97	<0.001
		Missing	119	6.5	40	33.6	63	52.9	16	13.4				
	Loneliness	No	1439	78.8	524	36.4	869	60.4	46	3.2	1.00			
		Yes	249	13.6	151	60.6	94	37.8	4	1.6	3.27	2.23	4.79	<0.001
		Missing	138	7.6	46	33.3	63	45.7	29	21.0				
	Uncertainty	No	778	42.6	239	30.7	499	64.1	40	5.1	1.00			
		Yes	996	54.5	468	47.0	499	50.1	29	2.9	2.89	1.93	4.31	<0.001
		Missing	52	2.8	14	26.9	28	53.8	10	19.2				

Legend: OR = Odds ratio; CI = Confidence Interval; Retir. = retired; Stud. = student; Housew. = housewife; behav. = behaviour Note: OR and CI were not calculated for missing values.

## Data Availability

The dataset generated and analyzed is retained by the Azienda Unità Sanitaria Locale–IRCCS di Reggio Emilia (Italy) and is available upon request from the corresponding author.

## Supplementary Materials

The following are available online at https://www.mdpi.com/1660-4601/18/1/306/s1, File S1: Survey on Lifestyle Adaptations in the General Population of the Province of Reggio Emilia Following the Emergency Quarantine COVID-19.

## Appendix A

**Table A1:** Results of Multivariate Regression Analysis Models, with Quality of Sleep as Dependent Variable and Financial Problems and Sociodemographic Characteristics as Independent Variables.

						95% Confidence Interval	
Predictor	Estimate	SE	Z	*p*	Odds Ratio	Lower	Upper
Intercept	−1.403	0.358	−3.923	<0 .001	0.246	0.122	0.496
**Sex**							
Male–Female	−0.840	0.264	−3.184	0.001	0.432	0.258	0.724
**Age class**							
Aged–Adult	−0.864	0.452	−1.911	0.056	0.421	0.174	1.023
Middle-aged–Adult	−0.141	0.191	−0.738	0.460	0.869	0.598	1.262
**Education level**							
High–Low	−1.073	0.350	−3.065	0.002	0.342	0.172	0.679
Medium–Low	−0.913	0.344	−2.656	0.008	0.401	0.205	0.787
**Financial problems**							
Many–None	1.940	0.376	5.156	<0 .001	6.962	3.329	14.556
Some–None	0.556	0.199	2.788	0.005	1.743	1.179	2.576

**Table A2:** Results of Multivariate Regression Analysis Models, with Quality of Sleep as Dependent Variable and Change in PA Habits and Sociodemographic Characteristics as Independent Variables.

						95% Confidence Interval	
Predictor	Estimate	SE	Z	*p*	Odds Ratio	Lower	Upper
Intercept	−1.245	0.352	−3.536	<0 .001	0.288	0.144	0.574
**Sex**							
Male–Female	−0.788	0.258	−3.061	0.002	0.455	0.274	0.753
**Age class**							
Aged–Adult	−0.985	0.452	−2.178	0.029	0.374	0.154	0.906
Middle-aged–Adult	−0.104	0.190	−0.544	0.586	0.902	0.621	1.309
**Education level**							
High–Low	−1.268	0.342	−3.709	<0.001	0.281	0.144	0.550
Medium–Low	−0.962	0.335	−2.871	0.004	0.382	0.198	0.737
**Change in PA habits**							
Improved–No change	−0.595	0.532	−1.120	0.263	0.551	0.195	1.563
Worsened–No change	0.483	0.183	2.638	0.008	1.621	1.132	2.321

Legend PA = Physical activity.

**Table A3:** Results of Multivariate Regression Analysis Models, with Quality of Sleep as Dependent Variable and Change in Eating Habits and Sociodemographic Characteristics as Independent Variables.

						95% Confidence Interval	
Predictor	Estimate	SE	Z	*p*	Odds Ratio	Lower	Upper
Intercept	−1.8735	0.395	−4.743	<0.001	0.154	0.0708	0.333
**Sex**							
Male–Female	−0.6464	0.253	−2.553	0.011	0.524	0.3190	0.861
**Age class**							
Aged–Adult	−0.5144	0.382	−1.347	0.178	0.598	0.2828	1.264
Middle-aged adult	−0.0689	0.187	−0.369	0.712	0.933	0.6476	1.346
**Education level**							
High–Low	−1.0647	0.340	−3.132	0.002	0.345	0.1771	0.671
Medium–Low	−0.8205	0.332	−2.474	0.013	0.440	0.2298	0.843
**Change in eating habits**							
Improved–No change	0.3883	0.268	1.450	0.147	1.474	0.8724	2.492
Worsened–No change	1.3103	0.266	4.930	<0.001	3.707	2.2019	6.242
Mixed–No change	0.8088	0.280	2.885	0.004	2.245	1.2960	3.890

**Table A4:** Results of Multivariate Regression Analysis Models, with Quality of Sleep as Dependent Variable and Feeling of Tension and Sociodemographic Characteristics as Independent Variables.

						95% Confidence Interval	
Predictor	Estimate	SE	Z	*p*	Odds Ratio	Lower	Upper
Intercept	−1.61890	0.371	−4.3681	<0.001	0.198	0.0958	0.410
**Sex**							
Male–Female	−0.74502	0.266	−2.8051	0.005	0.475	0.2821	0.799
**Age class**							
Aged–Adult	−0.40416	0.406	−0.9958	0.319	0.668	0.3013	1.479
Middle-aged adult	0.00382	0.190	0.0202	0.984	1.004	0.6924	1.455
**Education level**							
High–Low	−1.11701	0.359	−3.1102	0.002	0.327	0.1619	0.662
Medium–Low	−0.81351	0.353	−2.3049	0.021	0.443	0.2220	0.885
**Tension**							
Yes–No	1.25585	0.184	6.8324	<0.001	3.511	2.4488	5.033

**Table A5:** Results of Multivariate Regression Analysis Models, with Quality of Sleep as Dependent Variable and Feeling of Upset and Sociodemographic Characteristics as Independent Variables.

						95% Confidence Interval	
Predictor	Estimate	SE	Z	*p*	Odds Ratio	Lower	Upper
Intercept	−1.4162	0.367	−3.863	<0.001	0.243	0.118	0.498
**Sex**							
Male–Female	−0.8490	0.272	−3.116	0.002	0.428	0.251	0.730
**Age class**							
Aged–Adult	−0.6390	0.424	−1.507	0.132	0.528	0.230	1.212
Middle-aged Adult	−0.0489	0.190	−0.257	0.797	0.952	0.656	1.382
**Education level**							
High–Low	−1.2453	0.358	−3.483	<0.001	0.288	0.143	0.580
Medium–Low	−0.9358	0.352	−2.657	0.008	0.392	0.197	0.782
**Upset**							
Yes–No	1.1042	0.184	6.012	<0.001	3.017	2.105	4.324

**Table A6:** Results of Multivariate Regression Analysis Models, with Quality of Sleep as Dependent Variable and Feeling of Worry and Sociodemographic Characteristics as Independent Variables.

						95% Confidence Interval	
Predictor	Estimate	SE	Z	*p*	Odds Ratio	Lower	Upper
Intercept	−1.647	0.374	−4.41	<0 .001	0.193	0.0926	0.400
**Sex**							
Male–Female	−0.624	0.254	−2.46	0.014	0.536	0.3254	0.882
**Age class**							
Aged–Adult	−0.653	0.380	−1.72	0.086	0.520	0.2471	1.096
Middle-aged adult	−0.203	0.188	−1.08	0.280	0.817	0.5652	1.180
**Education level**							
High–Low	−1.093	0.350	−3.12	0.002	0.335	0.1688	0.666
Medium–Low	−0.796	0.343	−2.32	0.020	0.451	0.2305	0.883
**Worry**							
Yes–No	0.836	0.186	4.48	<0.001	2.306	1.6003	3.324

**Table A7:** Results of Multivariate Regression Analysis Models, with Quality of Sleep as Dependent Variable and Feeling of Fear and Sociodemographic Characteristics as Independent Variables.

						95% Confidence Interval	
Predictor	Estimate	SE	Z	*p*	Odds Ratio	Lower	Upper
Intercept	−1.309	0.365	−3.584	<0 .001	0.270	0.132	0.553
**Sex**							
Male–Female	−0.735	0.266	−2.760	0.006	0.480	0.285	0.808
**Age class**							
Aged–Adult	−0.773	0.422	−1.832	0.067	0.462	0.202	1.055
Middle-aged adult	−0.160	0.189	−0.847	0.397	0.852	0.588	1.234
**Education level**							
High–Low	−1.184	0.362	−3.268	0.001	0.306	0.150	0.623
Medium–Low	−0.904	0.356	−2.537	0.011	0.405	0.201	0.814
**Fear**							
Yes–No	0.884	0.195	4.536	<0.001	2.421	1.652	3.548

**Table A8:** Results of Multivariate Regression Analysis Models, with Quality of Sleep as Dependent Variable and Feeling of Loneliness and Sociodemographic Characteristics as Independent Variables.

						95% Confidence Interval	
Predictor	Estimate	SE	Z	*p*	Odds Ratio	Lower	Upper
Intercept	−1.354	0.374	−3.620	<0.001	0.258	0.124	0.538
**Sex**							
Male–Female	−0.836	0.264	−3.161	0.002	0.434	0.258	0.728
**Age class**							
Aged–Adult	−0.815	0.424	−1.923	0.054	0.443	0.193	1.016
Middle-aged adult	−0.140	0.192	−0.730	0.466	0.869	0.596	1.267
**Education level**							
High–Low	−1.153	0.368	−3.130	0.002	0.316	0.153	0.650
Medium–Low	−0.886	0.362	−2.448	0.014	0.412	0.203	0.838
**Loneliness**							
Yes–No	1.122	0.199	5.635	<0.001	3.070	2.079	4.536

**Table A9:** Results of Multivariate Regression Analysis Models, with Quality of Sleep as Dependent Variable and Feeling of Uncertainty and Sociodemographic Characteristics as Independent Variables.

						95% Confidence Interval	
Predictor	Estimate	SE	Z	*p*	Odds Ratio	Lower	Upper
Intercept	−1.874	0.383	−4.893	<0.001	0.154	0.0725	0.325
**Sex**							
Male–Female	−0.651	0.252	−2.577	0.010	0.522	0.3181	0.856
**Age class**							
Aged–Adult	−0.816	0.398	−2.051	0.040	0.442	0.2028	0.964
Middle-aged adult	−0.144	0.188	−0.769	0.442	0.866	0.5994	1.250
**Education level**							
High–Low	−1.119	0.351	−3.191	0.001	0.327	0.1643	0.649
Medium–Low	−0.770	0.341	−2.259	0.024	0.463	0.2375	0.903
**Uncertainty**							
Yes–No	1.000	0.206	4.853	<0.001	2.717	1.8146	4.069

Note. Estimates represent the log odds of “good sleep quality category” versus “poor sleep quality category”.
