# Supplementary material for: Poor Sleep during the First Peak of the SARS-CoV-2 Pandemic: A Cross-Sectional Study
Source: Int J Environ Res Public Health. 2021 Jan 4;18(1):306. doi: 10.3390/ijerph18010306 (PMC7795804; doi:10.3390/ijerph18010306)
Supplement: Supplementary file 1 [file ijerph-18-00306-s001.pdf]

Supplementary Material. Survey on Lifestyle Adaptations in the General Population of the Province of Reggio Emilia Following the Emergency Quarantine COVID-19.

Sociodemographic factors.

1. **Sex**

F	M

2. **Age**

18–23	24–29	30–35	36–41	42–47	48–53	54–59	60–65	66–71	>71

3. **City of residence: ___________________**
4. **Postal code: _________________**
5. **Citizenship:**
   - Italian
   - Foreign
6. **Marital status:**
   - Married
   - Cohabitation
   - Unmarried
   - Widowed
   - Divorced
   - Separated
7. **Education level**
   - None
   - Primary school
   - Middle high school
   - High school
   - University Degree
   - Post-university degree
8. **Current home size**
   - <50 m^2
   - 50 m^2–100 m^2
   - >100 m^2
9. **Does your home have any outdoor areas?**
   - Garden
   - Terrace
   - Balcony
   - Other
   - None
10. **Do you have any children?**
    - No

- Yes, at least one child under the age of 12
- Yes, all children over the age of 12

11. **Who do you live with? (multiple choice)**
    - Alone
    - With partner
    - With child/children
    - With parents
    - With a family member with a serious disability (child/parent)
    - With several family members with serious disabilities (children/parents)
    - With other relatives or cohabitants
12. **How many people live with you (excluding yourself): ________**
13. **Are you in isolation after testing positive for COVID-19?**
    - Yes
    - No
14. **If so, what kind of isolation?**
    - Own home
    - Hotel
    - Rems (COVID-19 residential facility)
    - Other temporary home

Work-related factors.

15. **Before the COVID-19 emergency, what was your employment status?**
    - Employed in the public sector
    - Employed in the private sector
    - Self-employed
    - Student
    - Retired
    - Unemployed
    - Housewife
    - Other (specify): ____________________
16. **Before the COVID-19 emergency, where did you work?**
    - At my workplace
    - From home
    - Both from home and at my workplace
17. **Current employment status**
    - Continue to work
    - On vacation leave
    - Leave of absence
    - Unemployment insurance

- Business closed due to the COVID-19 emergency
- Other

18. **If you work or study, how do you do these activities?**
    - My job has been suspended
    - I go to work
    - From home
    - I work both from home and at my workplace
    - I follow class lectures from home
19. **Are you currently experiencing economic problems?**
    - No problems
    - Some problems
    - Many problems

    Use of digital devices data.
20. **Do you currently have a suitable Internet connection?**
    - Yes
    - No
    - I don't know
21. **Do you currently have any IT devices, such as a computer, tablet, and/or smartphone, and a printer?**
    - Yes
    - No
    - I don't know
22. **How do you evaluate your computer literacy?**
    - Poor
    - Sufficient
    - Good
    - Excellent

    Lifestyle changes, anthropometric data, and health status.
23. **Weight:____**
24. **Height:______**
25. **Do you have any disease-related fee exemptions?**
    - Yes
    - No
26. **If so, for which disease?**
27. **Before the COVID-19 emergency, how did you consider yourself?**
    - Active (a person who has a physically demanding job that requires considerable physical effort and/or 30 min of moderate physical activity at least 5 days a week and/or

intense activity for more than 20 min for at least 3 days a week)

- Partially active (a person who does not do a physically demanding job but who does some physical activity in his/her free time)
- Sedentary (a person who neither has a physically demanding job nor does any physical activity in his/her free time)

28. **If you were active or partially active, how many days per week?**
    - 1–2 days/week
    - 3–4 days/week
    - >4 days/week
29. **Currently, how do you consider yourself?**
    - Active (a person who has a physically demanding job that requires considerable physical effort and/or 30 min of moderate physical activity at least 5 days a week and/or intense activity for more than 20 min for at least 3 days a week)
    - Partially active (a person who does not do a physically demanding job but who does some physical activity in his/her free time)
    - Sedentary (a person who neither has a physically demanding job nor does any physical activity in his/her free time)
30. **If you are active or partially active, how many days per week?**
    - 1–2 days/week
    - 3–4 days/week
    - >4 days/week
31. **What kind of physical activity do you currently do?**
    - Treadmill/running/walking/stairs
    - Stationary bike/bicycling
    - Pilates/yoga/stretching/free body/gym
    - Gardening
    - Other: ____________________
32. **What kind of IT do you use to perform physical activity at home?**
    - Group or one-to-one video chat
    - Online courses/guides
    - Applications on smartphones, tablets, computer
    - Programs from my trainer/coach
    - Alone
    - Other

33. **Has your diet changed (improved/worsened in terms of the consumption of some foods, mealtimes, weight control)?**
    - Yes
    - No
    - I don't know
34. **If so, what changes have occurred?**
    - I have add snacks, sweets, carbonated drinks
    - I eat more abundant main meals (breakfast and/or lunch and/or dinner)
    - I eat at more regular times
    - I pay more attention to eating healthier (i.e., quality and/or variety and/or meal cooking)
    - I eat more prepackaged foods
    - Before COVID-19, I was forced to often eat out
35. **Before the COVID-19 emergency, how many portions of fruit or vegetables did you eat daily?** (portion means a quantity of raw fruit or vegetables that can be held in the palm of your hand, or half a plate of cooked vegetables)
    - No portion of fruit or vegetables.
    - 1–2 portions/day
    - 3–4 portions/day
    - 5–6 portions/day
    - 6 portions/day
36. **Following the COVID-19 emergency, how has your consumption of fruit and vegetables changed?**
    - Increased
    - Decreased
    - Unchanged
    - I don't eat fruit or vegetables
37. **Do you currently consider your diet healthy?**
    - Yes
    - No
    - I don't know
38. **Before the COVID-19 emergency, how many alcoholic drinks did you consume?** (The alcoholic unit (UA) corresponds to 12 grams of ethanol, an amount approximately contained in a can of beer (330 mL at 4.5°), a glass of wine (125 mL at 12°), a glass of liqueur (40 mL at 40°), or an aperitif (80 mL at 38°)).
    - None
    - <2 alcoholic units (UA) on average per day
    - ≥2 alcoholic units (UA) on average per day

39. **Following the COVID-19 emergency, how has your consumption of alcohol changed?**

- Increased
- Decreased
- Unchanged
- I don't drink alcohol

40. **Before the emergency COVID-19, how did you consider yourself in terms of smoking?**

- Non-smoker (a person who claims to have smoked fewer than 100 cigarettes in his/her life (5 packs of 20) and is not currently a smoker)
- Smoker (a person who claims to have smoked at least 100 cigarettes in his/her life (5 packs of 20) and to be a smoker at the time of the interview or to have stopped smoking less than 6 months ago)
- Occasional smoker (a person who declares not to smoke every day)
- Daily smoker (a person who claims to smoke at least one cigarette every day).
- Ex-smoker (a person who claims to have smoked at least 100 cigarettes in his/her life (5 packs of 20), to NOT be a smoker at the time of the interview, and to have stopped smoking at least 6 months ago)
- Former smoker (a person who claims to have quit smoking less than 6 months ago)

41. **Following the COVID-19 emergency, how has your consumption of cigarettes changed?**

- Increased
- Decreased
- Unchanged
- I'm not a smoker

42. **Have your sleep habits changed during lockdown (sleep quantity and regularity)?**

- Yes
- No
- I don't know

43. **How do you consider your sleep quality now?**

- Very good
- Good
- Not very good
- Not good at all

44. **What are the new activities that you are doing that you did not do before COVID-19?**

- Reading
- Resting
- Cooking
- Watching TV
- Using social media
- Dedicating time to family activities
- Gardening / housecleaning
- Volunteer work
- None
- Other: ____________________

45. **In the future, are you going to maintain your acquired lifestyle habits or return to your previous behaviors?**

**(a) Physical activity**	maintain	return	I don't know/unchanged
**(b) Eating habits**	maintain	return	I don't know/unchanged
**(c) Smoking, drinking habits**	maintain	return	I don't know/unchanged
**(d) Sleep habits**	maintain	return	I don't know/unchanged
**(e) Leisure activities**	maintain	return	I don't know/unchanged

Use of the provincial support services network.

46. Have you used/Are you using any of the support made available by the municipality/associations/local health authorities?

- Home delivery of groceries
- Home delivery of medications
- Information hotline
- Mental health hotline
- Economic aid, e.g., food vouchers
- Area facilities for the care of animals
- Activation of social services
- Other: ____________________

47. **What kind of support would you have liked in order to deal with this situation but which was not available?**

____
____
____

48. **What/who helped you to overcome the challenges of lockdown?**

- family or friends
- volunteers/neighbors
- healthcare professionals
- the municipality or local associations

- leisure activities (i.e., reading, garden care, etc.)
- continuing to work
- optimistic attitude
- physical activity
- drinking and/or smoking
- taking care of pets
- salary
- drugs
- other

Emotional state.

49. At this moment, how are you feeling?

**Nervous**	1. Not at all	2. A little	3. Quite	4. A lot
**Upset**	1. Not at all	2. A little	3. Quite	4. A lot
**Worried**	1. Not at all	2. A little	3. Quite	4. A lot
**Afraid**	1. Not at all	2. A little	3. Quite	4. A lot
**Lonely**	1. Not at all	2. A little	3. Quite	4. A lot
**Uncertain about the future**	1. Not at all	2. A little	3. Quite	4. A lot
